# Adverse Pregnancy Outcomes and Maternal Periodontal Disease: An Overview on Meta-Analytic and Methodological Quality

**DOI:** 10.3390/jcm12113635

**Published:** 2023-05-23

**Authors:** Vanessa Machado, Madalena Ferreira, Luísa Lopes, José João Mendes, João Botelho

**Affiliations:** 1 Clinical Research Unit (CRU), Egas Moniz Center for Interdisciplinary Research, Egas Moniz School of Health and Science, 2829-511 Caparica, Portugal; 2 Evidence-Based Hub, Egas Moniz Center for Interdisciplinary Research, Egas Moniz School of Health and Science, 2829-511 Almada, Portugal

**Keywords:** adverse pregnancy outcomes, oral health, periodontitis, umbrella review

## Abstract

This umbrella review aims to appraise the methodological quality and strength of evidence on the association between maternal periodontitis and adverse pregnancy outcomes (APOs). PubMed, CENTRAL, Web-of-Science, LILACS, and Clinical Trials were searched until February 2023, without date or language restrictions. Two authors independently screened studies, extracted data, performed the risk-of-bias analysis, and estimated the meta-analytic strengths and validity and the fail-safe number (FSN). A total of 43 SRs were identified, of which 34 conducted meta-analyses. Of the 28 APOs, periodontitis had a strong association with preterm birth (PTB), low birth weight (LBW), and gestational diabetes mellitus (GDM), PTB and LBW showed all levels of strength, and pre-eclampsia showed only suggestive and weak strength. Regarding the consistency of the significant estimates, only 8.7% were likely to change in the future. The impact of periodontal treatment on APOs was examined in 15 SRs, 11 of which conducted meta-analyses. Forty-one meta-analyses were included and showed that periodontal treatment did not have a strong association with APOs, although PTB revealed all levels of strength and LBW showed only suggestive and weak evidence. Strong and highly suggestive evidence from observational studies supports an association of periodontitis with a higher risk of PTB, LBW, GDM, and pre-eclampsia. The effect of periodontal treatment on the prevention of APOs is still uncertain and requires future studies to draw definitive and robust conclusions.

## 1. Introduction

Periodontitis is a chronic disease characterized by persistent inflammation that progressively damages the tissues surrounding the teeth [1,2]. The homeostasis disruption results in a host immune and inflammatory response in the periodontium with tissue destruction, gingival bleeding, and systemic inflammatory repercussions [1,2]. Among the several systemic conditions linked to periodontitis, adverse pregnancy outcomes (APOs) stand out [3,4,5] with their impact on maternal and infant health.

Approximately 40% of pregnant women worldwide are estimated to suffer from periodontitis [3,6]. During pregnancy, hormonal changes promote vascular permeability, which increases the likelihood of gingival inflammation [5,7]. The oral microbiome of pregnant women is a relatively stable community [8], but it can shift to distinct compositions [9,10,11] that may increase the risk of periodontitis [7] and, consequently, the association withother maternal complications such as gestational diabetes mellitus (GDM) [12]. Yet, prenatal dental care effectively reduces the carriage of oral pathogens (such as *Streptococcus mutans*) [8].

Since Offenbacher et al. in 1996 first reported a possible association between periodontal disease and preterm birth (PTB) [13], a new series of studies in periodontal medicine has linked maternal periodontitis to APOs, namely PTB, low birth weight (LBW), pre-eclampsia, GDM, and miscarriage/stillbirth (M/SB) [4,14].

With the exponential increase in clinical studies, a large number of systematic reviews regarding this association have emerged. In the light of current knowledge, five previous umbrella reviews summarized the available evidence and identified gaps in this association [15,16,17,18,19]. Nevertheless, none of the latter explored the statistical consistency of the estimates in the context of the available research. Therefore, an umbrella review assessing the methodological quality and strength of evidence on the association between maternal periodontitis and APOs was deemed timely.

## 2. Materials and Methods

This umbrella review was defined a priori by all authors, published online in the PROSPERO platform (ID: CRD42022358842), and conducted according to the Preferred Reporting Items for Systematic Reviews and Meta-Analysis (PRISMA) guidelines (Supplementary Data S1) [20].

### 2.1. Focused Question and Eligibility Criteria

The following focused PI(E)CO questions were addressed: “Do women with periodontitis have an increased risk of APOs compared to women without periodontitis?” (Population: Pregnant women; Exposure: Periodontitis; Comparison: Non-periodontitis; Outcomes: APOs) and “Does periodontal treatment reduce the risk of APOs?” (Population: Pregnant women; Intervention: Periodontal treatment; Comparison: No periodontal treatment; Outcomes: APOs).

Studies were eligible for inclusion based on the following criteria: (1) systematic reviews with or without meta-analysis; (2) retrieved data from human studies; (3) evaluated the association between APOs and periodontal disease (either observational or interventional). There were no restrictions regarding the year or language of publication. Exclusion criteria were as follows: (1) systematic reviews of systematic reviews (umbrella reviews); (2) commentaries, abstracts, letters to the editor, or consensus; (3) unsuitable inclusion criteria; and (4) inclusion of animal studies in the meta-analysis.

### 2.2. Study Selection

We conducted a comprehensive search of the following five electronic databases: PubMed (via MEDLINE), Cochrane Database of Systematic Reviews, Web of Science (WOS), Latin-American Scientific Literature in Health Sciences (LILACS), and Clinical Trials.gov, from the earliest data available up to February 2023. We merged keywords and subject headings according to the thesaurus of each database: #1: (periodontal diseases[MeSH]) OR (gingivitis[MeSH]) OR (gingival inflammation) OR (periodontal health) OR (root planing[MeSH]) OR (periodontal therapy) OR (periodontal treatment) OR (scaling and root planing) OR (supragingival and subgingival scaling); #2: (Pregnant Women[MeSH]) OR (Pregnancy[MeSH]) OR (Parturition[MeSH]); #3: (pregnancy outcome[MeSH])) OR (pregnancy complications[MeSH]) OR (premature birth[MeSH]) OR (low birth weight) OR (gestational Age[MeSH]) OR (Diabetes, Gestational[MeSH]) OR (Abortion, Spontaneous[MeSH]); #3: (systematic review) OR (meta-analysis) OR (metaanalysis); #1 AND #2 AND #3. The search was adapted according to each database, using the same keywords and word combinations. Additional relevant literature was included after a manual search of six periodontology- and gynecology-specific journals (namely, Obstetrics and Gynecology, British Journal of Obstetrics and Gynecology, American Journal of Obstetrics and Gynecology, Journal of Periodontal Research, Journal of Clinical Periodontology, and Journal of Periodontology). The grey literature was searched using the OpenGrey portal (http://www.opengrey.eu/ (accessed on 23 February 2023)). The electronic search was performed by two researchers (M.F. and V.M.) who independently screened the titles and abstracts of all the retrieved articles and excluded duplicates and unrelated studies. Any disagreements were resolved by discussion with a third reviewer (J.B.).

### 2.3. Data Items and Data Collection Process

A predefined table was used to extract the necessary data from each eligible study, including study identification (authors, publication year, country of origin), search period, number and type of the included studies, population size, periodontal case definition and clinical measures, obstetric complication outcomes, methodological quality tool used, effect size and 95% CI, and funding information. All information was extracted by two independent researchers (M.F. and V.M.), and any disagreements were resolved by discussion with a third researcher (J.B.). Intra- and inter-examiner agreement was assessed using Cohen’s Kappa statistic (0.89; 95% CI: 0.87–0.90). Corresponding authors were contacted, when necessary to clarify data or obtain missing information.

### 2.4. Methodological Quality Appraisal

The included systematic reviews were independently assessed by two reviewers (M.F. and V.M.) using the A MeaSurement Tool to Assess Systematic Reviews (AMSTAR 2) [21]. AMSTAR 2 is a comprehensive 16-item tool that rates the overall confidence in the results of the review. According to the AMSTAR 2 guidelines, systematic reviews are categorized as: High (“zero or one non-critical weakness”); Moderate (“more than one non-critical weakness”); Low (“one critical flaw with or without non-critical weaknesses”); and Critically Low (“more than one critical flaw with or without non-critical weaknesses”).

### 2.5. Meta-Analytical Estimates Strengths and Validity

Data were processed and managed using Excel from MS Office 365 to calculate inferential statistical analyses. To grade meta-analyses, we used a previously defined methodology by Papadimitrou et al. (2021) [22]. Therefore, associations were defined into four levels of evidence: strong, highly suggestive, suggestive, and weak evidence [22,23], as follows:

- Strong evidence: >1000 cases included in the meta-analysis, based on a threshold that ensured 80% power for hazard ratios ≥ 1.20 (α = 0.05) 38; *p*-value ≤ 10^−6^ of statistical significance in meta-analysis 50–52; heterogeneity (I^2^) below 50%; the null value was excluded by the 95% prediction interval; and no evidence of small study effects or excess significance bias.
- Highly suggestive: >1000 cases were included in the meta-analysis; *p*-value ≤ 10^−6^, and the largest study in the meta-analysis was statistically significant.
- Suggestive evidence: >1000 cases were included in the meta-analysis, and random effects ≤ 10^−3^ 50–52 was categorized.
- Weak evidence: if the latter conditions were not verified.

The fail-safe number (FSN) for statistically significant meta-analyses was then calculated using Rosenberg’s FSN [24], followed by the median and range for each evidence grade (strong, highly suggestive, suggestive, and weak).

### 2.6. Overlap

Total overlap according to the association of periodontal disease with APOs or the effect of periodontal therapy on APOs was determined using the formula proposed by Pieper et al. [25]. The results were expressed as percentages and corrected covered area (CCA) values between 0 and 15. A CCA value of 0–5 indicates low overlap, 6–10 moderate overlap, 11–15 high overlap, and >15 very high overlap.

## 3. Results

### 3.1. Study Selection and Systematic Reviews Characteristics

**Figure 1:**
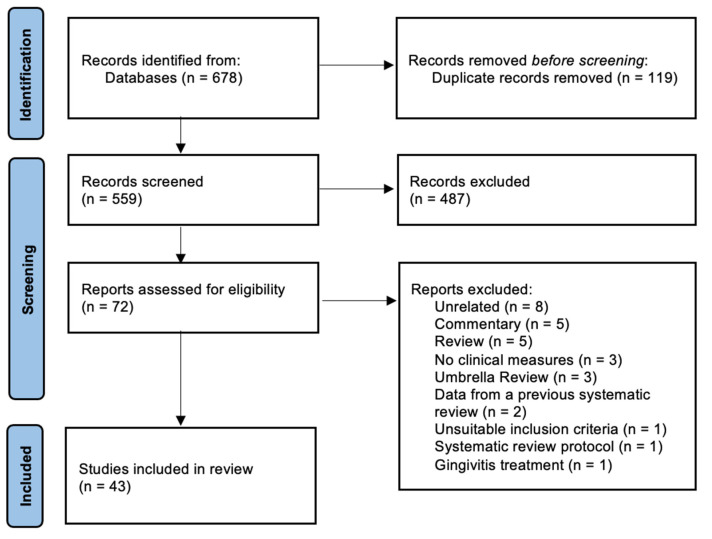
PRISMA flowchart showing the exclusion and inclusion process of the literature review.

The search strategy yielded a total of 678 potentially relevant studies (Figure 1). After removing duplicates (n = 119), a total of 559 records were screened for eligibility criteria by title and abstract, and 487 records were excluded. After full-paper assessment, 29 were excluded with the respective reasons for exclusion detailed in Supplementary Data S2. As a result, 43 systematic reviews met all of the eligibility criteria and were included for qualitative synthesis and 19 for quantitative analyses.

Overall, we included 34 and 9 systematic reviews with and without meta-analysis, respectively (Supplementary Data S3). Of the 43 articles analyzed, 37.2% (n = 16) were published in dental journals, 32.6% (n = 14) in obstetrics and gynecology journals, and 30.2% (n = 13) in general medical journals. Almost 40% (n = 17) of all the included articles were published between 2010 and 2013. Regarding study type, 16 systematic reviews included only controlled trials, 20 included only observational studies, and 7 included both. Twenty-eight systematic reviews addressed the association between maternal periodontal status and the risk of APOs, while the other 15 studies aimed to evaluate the impact of periodontal treatment during pregnancy on perinatal outcomes. The majority followed the PRISMA guidelines (39.5%, n = 17), although 39.5% (n = 17) did not prepare the review process according to any standardized guideline (Supplementary Data S3). Regarding methodological quality assessment, Cochrane tools (27.9%, n = 12) and the Newcastle-Ottawa scale (23.3%, n = 10) were the most commonly used instruments.

### 3.2. Methodological Quality Assessment

**Figure 2:**
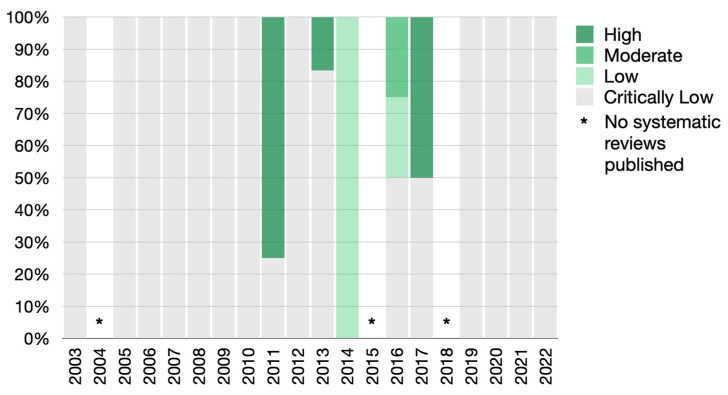
Diagram showing results from the methodological quality assessment of included systematic reviews.

Good inter-examiner reliability was found for the AMSTAR 2 screening (Cohen Kappa score  =  0.84; 95% CI: 0.81–0.88). Overall, 35 studies were judged to be of critically low quality, 2 as of low quality, 1 as of moderate quality and 5 of high methodological quality (Supplementary Data S3, Figure 2 and detailed in Supplementary Data S4). Of those of high quality, 2 studies explored the association of periodontal disease with APOs and the remaining 3 studies investigated the effect of periodontal treatment on APOs. Only one of the included systematic reviews fully met the AMSTAR-2 checklist. Regarding language restriction, 7 systematic reviews did not report this characteristic, 18 applied a language restriction, and the remaining 18 studies had no language restrictions. Furthermore, most systematic reviews failed to report the sources of funding for the studies included in the review (90.7%, n = 39), to search for grey literature (86.0%, n = 37), to specify a plan to investigate causes of heterogeneity (65.1%, n = 28), to search trial and/or study registries (58.1%, n = 25), and to provide a list of excluded studies with justification (53.5%, n = 23). Study selection and data extraction in duplicates were not performed in 20.9% (n = 9) and 32.6% (n = 14), respectively. In addition, the definition of the review methods a priori was considered in only 5 studies (11.6%) for all included systematic reviews.

### 3.3. The Association of Periodontal Disease with APOs

Overall, 28 systematic reviews analyzed the association of periodontal disease with APOs, of which 14 systematic reviews conducted meta-analyses (53.8%). A total of 28 meta-analytical estimates were conducted and analyzed below. The overlap observed was58.7% with a CCA of 5.74% (Supplementary Data S5).

#### 3.3.1. Meta-Analytic Strength of Estimates

**Figure 3:**
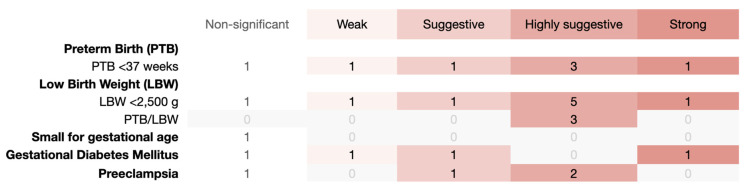
Evidence grading map of systematic reviews on the association of periodontal disease with adverse pregnancy outocmes (APOs). The information at the top of the map shows the evidence grading scale (from left to right, increasing the evidence level). At the left side of the map, each APO (with detailed information) is presented.

Of the twenty-eight estimates, periodontal disease had a strong association with three APOs: PTB (less than 37 weeks), LBW (less than 2500 g), and gestational diabetes mellitus (Figure 3). PTB (less than 37 weeks) and LBW (less than 2500 g) showed all the remaining levels of strength, yet pre-eclampsia showed suggestive and weak evidence. In addition, highly suggestive evidence was found for the combination PTB/LBW (n = 3). Small for gestational age was the only APO that did not show significance of association.

#### 3.3.2. Consistency of Evidence

When analyzing the consistency of the significant estimates (weak to strong) shown in Figure 3, only 8.7% of the estimates (2 out of 23) were likely to change in the future, according to the FSN statistics, indicating a fairly robust consistency (Supplementary Data S3). Of these two, both were classified as weak meta-analyses for PTB (less than 37 weeks) and LBW (less than 2500 g). None of the strong meta-analytic estimates had the potential to change in the future, indicating consistency.

### 3.4. Periodontal Treatment Effect on APOs

A total of fifteen systematic reviews analyzed the effect of periodontal treatment on APOs, of which 11 conducted meta-analyses (73.3%). A total of 41 meta-analytical estimates were conducted and analyzed below. Overlap was 77.4%, with a CCA of 33.4% (Supplementary Data S6).

#### 3.4.1. Meta-Analytic Strength of Estimates

**Figure 4:**
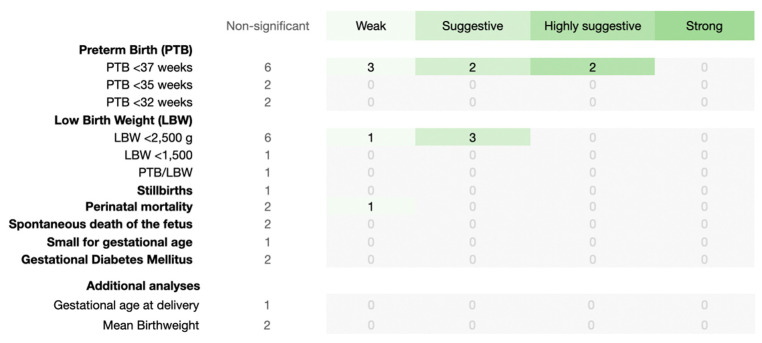
Evidence grading map from a meta-analysis studying the association of periodontal treatment with adverse pregnancy outcomes (APOs). The information at the top of the map shows the evidence grading scale (from left to right, increases the evidence level). On the left side of the map, each APO (with detailed information) is presented.

Of the 41 estimates, periodontal treatment did not have a strong association with APOs (Figure 4). Nevertheless, PTB (less than 37 weeks) showed all the remaining levels of strength, but LBW (less than 2500 g) showed only suggestive and weak evidence. In addition, perinatal mortality was the only APO that showed weak significancant association. The remaining APOs showed no significant association with periodontal treatment.

#### 3.4.2. Consistency of Evidence

When analyzing the consistency of the generated estimates shown in Figure 4, 58.3% of the estimates (7 out of 12) were likely to change in the future, according to the FSN statistics, indicating a little robust consistency (Supplementary Data S3). Of these seven, four related to PTB (less than 37 weeks) (one highly suggestive, one suggestive, and two weak), two suggestive meta-analyses for LBW (less than 2500 g), and one weak meta-analysis for perinatal mortality (Supplementary Data S3).

## 4. Discussion

### 4.1. Main Findings

The present umbrella review evaluated a total of 43 systematic reviews with a total sample of 67 meta-analytic comparisons to assess the quality of evidence in two main categories: (i) pregnant women with periodontitis have an increased risk of APO; and (ii) periodontal treatment effects on APO. Three associations were supported by strong meta-analytic evidence, endorsing highly significant results with no suggestive bias. These associations were between periodontitis diagnosis and a higher risk of LBW (less than 2500 g) [26] and PTB (less than 37 weeks) [4], as well as GDM [27]. Fourteen associations were supported by highly suggestive evidence, most involving periodontitis and a higher risk of LBW (less than 2500 g) [5,26,28,29], PTB (less than 37 weeks) [5,28,29], PTB/LBW [28,29,30], and pre-eclampsia [31,32]. Additionally, periodontal treatment was inversely associated with the risk of PTB (less than 37 weeks) [33,34].

Several factors have been clearly associated with the risk of LBW, PTB, pre-eclampsia, and GDM. Although periodontitis-related factors are an established risk for increasing systemic inflammatory burden, the association of periodontitis with APO risk is less known and potentially biased due to exposure measurement error and reporting bias. To overcome this obstacle, we used statistical tests and sensitivity analyses to search for evidence of bias. A total of 71 meta-analyses were evaluated, but on average, they contained relatively few studies (median = 8). Almost 52% (n = 37) of the included associations between periodontitis diagnosis or treatment and APOs risk reported a statistically significant summary random-effects estimate. Additionally, this proportion of significant associations decreased to 25.4% (n = 18) when a lower *p* value threshold (*p* < 10^−6^) was used, pointing to a lack of existing robust associations. One in three associations showed high levels of heterogeneity (I^2^ ≥ 50%). Moreover, when the FSN research method was used to consider observational studies, the majority of studies (21 out of 23) were unlikely to change the current evidence for associations. Otherwise, when the FSN was calculated in interventional studies, more than 58.3% (7 out of 12) were likely to change the existing research.

The present umbrella supports the notion that there are a limited number of periodontal-related factors and periodontal treatment follow-up data that are robustly associated with APOs risk. Nevertheless, it is critical to continue and increase research efforts in this field because APOs can be life-threatening to both the mother and the fetus/baby.

### 4.2. Agreement and Disagreement with Previous Umbrella Reviews

To the best of the authors’ knowledge, eight umbrella reviews addressed and summarized the available evidence in this association between periodontitis and APOS, and the effect of periodontal disease on pregnancy complications [15,16,17,18,19,35,36,37]. In all eight published umbrella reviews, the authors only analyzed and interpreted the methodological quality and described the main findings of the included systematic reviews on maternal periodontitis and APOs. This umbrella review goes beyond these very basic aims of a systematic review. We have analyzed the meta-analytic estimates of all systematic reviews with meta-analyses and provide definitive conclusions on whether future research is likely to change the results of existing significant meta-analyses. Briefly stated, we provide solid and enduring evidence maps that will decisively contribute to draw oral and periodontal care strategies for pregnant women with the primary goal of minimizing pregnancy complications. This, to the best of our knowledge, has no equivalent umbrella review conclusion.

Regarding the methodological quality, four of five umbrella reviews used AMSTAR-1 (the first version of this tool, published in 2007), whereas only two umbrella reviews [35,37] used AMSTAR-2 as we did. Furthermore, Condylis et al. [15] did not assess the methodological quality of the evidence of the included studies at all, which can be considered a serious methodological flaw. Lavigne et al. [36] used the PRISMA checklist to assess the quality of systematic reviews, although this checklist was not designed for this purpose.

This umbrella review analyzes the evidence that has been produced in this field. We did not limit our search to a specific area (observational or interventional) or time period. Therefore, we have results from over six APOs associations with periodontitis diagnosis and thirteen effects of periodontal treatment in APOs. In contrast, only one umbrella review [19] examined the association of periodontitis with three APOs (LBW, PTB, and pre-eclampsia) through observational studies, in contrast to ours (six in total: PTB and LBW [both individual or combined outcomes], small for gestational age, gestational diabetes mellitus, and pre-eclampsia). Furthermore, while the previous umbrella reviews examined the effect of periodontal therapy on a few specific APOs (PTB [<37 weeks; <35 weeks], LBW and pre-eclampsia), ours explored this effect on thirteen APOs (PTB [<37 weeks; <35 weeks and <32 weeks], LBW [<2500 g and <1500 g], stillbirth, spontaneous fetal death, small for gestational age, gestational diabetes mellitus, and additional analyses such as gestational age at delivery and mean birth weight).

Overall, 44 systematic reviews from inception to February 2023 were included, while Condylis et al. [15] included 15 RCTs and 5 meta-analyses published until January 2011, Lopez et al. [16] included 6 systematic reviews published between 2003 and March 2012, Vilares-Builes et al. [19] included 19 systematic reviews and did not report the search period, Rangel-Ricon et al. [18] included 18 systematic reviews, and Matei et al. [17] included 9 studies in the periodontal field published between January 2005 and October 2016. Notwithstanding, we included 18 systematic reviews that were not included in previously published umbrella reviews, accounting for nearly 40% of all available meta-evidence.

### 4.3. Strengths and Limitations

This umbrella review presents strengths and shortcomings worth discussing to help readers interpret these findings. First, it provides a comprehensive overview of the available systematic reviews on the relationship between maternal periodontitis and APOs, following a strict protocol with a transparent and evidence-based methodology. Second, it presents an evidence grid map from nonsignificant to strong associations and distributes information by APO. Third, this umbrella review goes beyond the classical approach by attempting to explore whether the current evidence is likely to change using an FSN methodology. In other words, the application of these metrics demonstrates whether or not further research can influence the existing meta-analytic evidence and therefore provides guidance for future research agendas and public health policy. However, readers must also be aware of the limitations of the FSN methodology. The FSN represents the number of studies required to refute a significant meta-analytic mean, and is purely mathematical estimate, focusing on whether the *p*-value reaches an arbitrary threshold. It is also highly dependent on the assumed mean intervention effect of the unpublished studies. In this respect, the level of evidence appears to be much more consistent at the observational level (8.7% of estimates are likely to change with future research) than at the interventional level (58.3% of estimates are likely to change with future research).

Nevertheless, the majority of the included systematic reviews are of critically low quality (high risk of bias), and this is the most relevant shortcoming of this review. This conclusion is based on the methodological quality assessment conducted with AMSTAR-2 and will guide future evidence-based research. In addition, this review makes clear that most evidence is based on observational data with a low percentage of longitudinal studies and randomized trials. Additionally, consideration of overlap is critical when conducting systematic reviews [25]. We observed moderate and high levels of overlap for observational and interventional studies, respectively. To address this issue, we presented the overlap as a percentage and the CCA for each outcome and comparison. Hence, the current evidence is based upon more non-inferential evidence than definitive causal assumptions. A further shortcoming is that almost all meta-analyses produced unmeasured estimates for confounding variables, and it is therefore recommended that future studies report effect sizes adjusted for confounding factors.

### 4.4. Implications for Practice and Research

The results of this umbrella review highlight the importance of periodontal health during pregnancy for the systemic health of both mother and child. The relationship between maternal periodontal status and the risk of APOs, namely PTB, LBW, and GDM, has been demonstrated by a large body of evidence. However, pregnant women do not seek dental care [38] due to unawareness, fear, and/or lack of access. Obstetricians-gynecologists and other obstetric care providers (e.g., nurses, midwives) can play a critical role in changing this paradigm, as they are the health professionals who maintain the closest contact with expectant mothers. For this reason, it is very important that articles on this topic be published in obstetric and gynecologic journals. This information is extremely relevant and allows for a more holistic approach to pregnancy and the integration of oral health programs into prenatal care.

These findings also emphasize that more studies are warranted to further investigate the interplay between maternal periodontitis and APOs, as well as its underlying biological mechanisms. Additionally, the level of evidence found in this umbrella makes clear the need for more trials and increased patient data to face the uncertainty of meta-analytic estimates published so far. Therefore, further exploration of the clinical efficacy of periodontal therapy before and during pregnancy is still a necessary topic of research through intervention studies. The use of the new 2018 Classification for Periodontal and Peri-implant Diseases [39] is highly recommended to improve the standardization of studies and allow future conclusions on this association.

## 5. Conclusions

Strong and highly suggestive evidence from observational studies supports an association of periodontitis with an increased risk of PTB, LBW, GDM, and pre-eclampsia. Additional similar research is unlikely to change the current evidence for the association between periodontitis status and APOs, with few exceptions, indicating robust consistency. The level of evidence on the effect of periodontal treatment on the prevention of APOs is still uncertain and requires future studies to draw definitive and robust conclusions. These results strongly recommend periodontal primary prevention care as a key health standard in prenatal and perinatal care programs.

## Supplementary Materials

The following supporting information can be downloaded at: https://www.mdpi.com/article/10.3390/jcm12113635/s1, Supplementary Data S1. PRISMA Checklist; Supplementary Data S2. List of excluded studies with justification for exclusion.; Supplementary Data S3. Characteristics of the included studies. Supplementary Data S4. AMSTAR 2 results; Supplementary Data S5. Overlap of study results across systematic reviews on the association of periodontal disease with APOs; Supplementary Data S6. Overlap of study results across systematic reviews on the periodontal treatment effect on APOs Refs. [40,41,42,43,44,45,46,47,48,49,50,51,52,53,54,55,56,57,58,59,60,61,62,63,64,65,66,67,68] are cited in the Supplementary Materials.
