# Supplementary material for: Adverse Pregnancy Outcomes and Maternal Periodontal Disease: An Overview on Meta-Analytic and Methodological Quality
Source: J Clin Med. 2023 May 23;12(11):3635. doi: 10.3390/jcm12113635 (PMC10253546; doi:10.3390/jcm12113635)
Supplement: Supplementary file 1 [file jcm-12-03635-s001.zip › jcm-2286740-supplementary.pdf]

An umbrella review of the evidence linking adverse pregnancy outcomes and maternal periodontal disease: from the observation to clinical intervention

Online Supplemental Information

Supplementary data 1. PRISMA Checklist.

Section and Topic	Item #	Checklist item	Location where item is reported
**TITLE**			
Title	1	Identify the report as a systematic review.	1
**ABSTRACT**			
Abstract	2	See the PRISMA 2020 for Abstracts checklist.	5
**INTRODUCTION**			
Rationale	3	Describe the rationale for the review in the context of existing knowledge.	7
Objectives	4	Provide an explicit statement of the objective(s) or question(s) the review addresses.	8
**METHODS**			
Eligibility criteria	5	Specify the inclusion and exclusion criteria for the review and how studies were grouped for the syntheses.	8-9
Information sources	6	Specify all databases, registers, websites, organisations, reference lists and other sources searched or consulted to identify studies. Specify the date when each source was last searched or consulted.	9
Search strategy	7	Present the full search strategies for all databases, registers and websites, including any filters and limits used.	9
Selection process	8	Specify the methods used to decide whether a study met the inclusion criteria of the review, including how many reviewers screened each record and each report retrieved, whether they worked independently, and if applicable, details of automation tools used in the process.	10
Data collection process	9	Specify the methods used to collect data from reports, including how many reviewers collected data from each report, whether they worked independently, any processes for obtaining or confirming data from study investigators, and if applicable, details of automation tools used in the process.	10
Data items	10a	List and define all outcomes for which data were sought. Specify whether all results that were compatible with each outcome domain in each study were sought (e.g. for all measures, time points, analyses), and if not, the methods used to decide which results to collect.	10
	10b	List and define all other variables for which data were sought (e.g. participant and intervention characteristics, funding sources). Describe any assumptions made about any missing or unclear information.	10
Study risk of bias assessment	11	Specify the methods used to assess risk of bias in the included studies, including details of the tool(s) used, how many reviewers assessed each study and whether they worked independently, and if applicable, details of automation tools used in the process.	10-11
Effect measures	12	Specify for each outcome the effect measure(s) (e.g. risk ratio, mean difference) used in the synthesis or presentation of results.	10-11
Synthesis methods	13a	Describe the processes used to decide which studies were eligible for each synthesis (e.g. tabulating the study intervention characteristics and comparing against the planned groups for each synthesis (item #5)).	11-12
	13b	Describe any methods required to prepare the data for presentation or synthesis, such as handling of missing summary statistics, or data	11-12

Section and Topic	Item #	Checklist item	Location where item is reported
		conversions.	
	13c	Describe any methods used to tabulate or visually display results of individual studies and syntheses.	11-12
	13d	Describe any methods used to synthesize results and provide a rationale for the choice(s). If meta-analysis was performed, describe the model(s), method(s) to identify the presence and extent of statistical heterogeneity, and software package(s) used.	NA
	13e	Describe any methods used to explore possible causes of heterogeneity among study results (e.g. subgroup analysis, meta-regression).	NA
	13f	Describe any sensitivity analyses conducted to assess robustness of the synthesized results.	NA
Reporting bias assessment	14	Describe any methods used to assess risk of bias due to missing results in a synthesis (arising from reporting biases).	NA
Certainty assessment	15	Describe any methods used to assess certainty (or confidence) in the body of evidence for an outcome.	11-12
**RESULTS**			
Study selection	16a	Describe the results of the search and selection process, from the number of records identified in the search to the number of studies included in the review, ideally using a flow diagram.	12-13
	16b	Cite studies that might appear to meet the inclusion criteria, but which were excluded, and explain why they were excluded.	12
Study characteristics	17	Cite each included study and present its characteristics.	12-13
Risk of bias in studies	18	Present assessments of risk of bias for each included study.	13
Results of individual studies	19	For all outcomes, present, for each study: (a) summary statistics for each group (where appropriate) and (b) an effect estimate and its precision (e.g. confidence/credible interval), ideally using structured tables or plots.	14-15
Results of syntheses	20a	For each synthesis, briefly summarise the characteristics and risk of bias among contributing studies.	14-15
	20b	Present results of all statistical syntheses conducted. If meta-analysis was done, present for each the summary estimate and its precision (e.g. confidence/credible interval) and measures of statistical heterogeneity. If comparing groups, describe the direction of the effect.	NA
	20c	Present results of all investigations of possible causes of heterogeneity among study results.	NA
	20d	Present results of all sensitivity analyses conducted to assess the robustness of the synthesized results.	NA
Reporting biases	21	Present assessments of risk of bias due to missing results (arising from reporting biases) for each synthesis assessed.	NA
Certainty of evidence	22	Present assessments of certainty (or confidence) in the body of evidence for each outcome assessed.	14-15
**DISCUSSION**			

Section and Topic	Item #	Checklist item	Location where item is reported
Discussion	23a	Provide a general interpretation of the results in the context of other evidence.	11
	23b	Discuss any limitations of the evidence included in the review.	15
	23c	Discuss any limitations of the review processes used.	15
	23d	Discuss implications of the results for practice, policy, and future research.	17
**OTHER INFORMATION**			
Registration and protocol	24a	Provide registration information for the review, including register name and registration number, or state that the review was not registered.	16-17
	24b	Indicate where the review protocol can be accessed, or state that a protocol was not prepared.	8
	24c	Describe and explain any amendments to information provided at registration or in the protocol.	NA
Support	25	Describe sources of financial or non-financial support for the review, and the role of the funders or sponsors in the review.	1
Competing interests	26	Declare any competing interests of review authors.	1
Availability of data, code and other materials	27	Report which of the following are publicly available and where they can be found: template data collection forms; data extracted from included studies; data used for all analyses; analytic code; any other materials used in the review.	NA

From: Page MJ, McKenzie JE, Bossuyt PM, Boutron I, Hoffmann TC, Mulrow CD, et al. The PRISMA 2020 statement: an updated guideline for reporting systematic reviews. BMJ 2021;372:n71. doi: 10.1136/bmj.n71

For more information, visit: http://www.prisma-statement.org/

Supplementary data 2. List of excluded studies with justification for exclusion.

N		Reasons to exclude
1	Gharehghani MAM, Bayani A, Bayat AH, Hemmat M, Karimy M, Ahounbar E, Armoon B, Fakhri Y, Schroth RJ. Poor oral health-related quality of life among pregnant women: A systematic review and meta-analysis. Int J Dent Hyg. 2021 Feb;19(1):39-49. doi: 10.1111/idh.12465. Epub 2020 Oct 4. PMID: 32941664.	Unsuitable inclusion criteria
2	Bhola S, Geddis-Regan A. Could optimising periodontal health in expectant mothers reduce the risk of babies being born prematurely? Evid Based Dent. 2021 Jan;22(1):14-15. doi: 10.1038/s41432-021-0149-3. PMID: 33772122.	Commentary
3	Turcu-Duminică, Ana; Dumitriu, Anca Silvia; Paunica, Stana; Gică, Corina; Botezatu, Radu; Gică, Nicolae; Peltecu, Gheorghe; and Panaitescu, Anca Maria (2021) "Periodontitis as a potential risk factor for premature delivery," *Journal of Mind and Medical Sciences*: Vol. 8: Iss. 1, Article 5.	Review
4	Le QA, Eslick GD, Coulton KM, Akhter R, Condous G, Eberhard J, Nanan R. Does Treatment of Gingivitis During Pregnancy Improve Pregnancy Outcomes? A Systematic Review and Meta-Analysis. Oral Health Prev Dent. 2021 Jan 7;19(1):565-572. doi: 10.3290/j.ohpd.b2183059. PMID: 34673848.	Gingivitis treatment
5	Favero V, Bacci C, Volpato A, Bandiera M, Favero L, Zanette G. Pregnancy and Dentistry: A Literature Review on Risk Management during Dental Surgical Procedures. Dent J (Basel). 2021 Apr 19;9(4):46. doi: 10.3390/dj9040046. PMID: 33921608; PMCID: PMC8072957.	Review
6	Jakovljevic A, Sljivancanin Jakovljevic T, Duncan HF, Nagendrababu V, Jacimovic J, Aminoshariae A, Milasin J, Dummer PMH. The association between apical periodontitis and adverse pregnancy outcomes: a systematic review. Int Endod J. 2021 Sep;54(9):1527-1537. doi: 10.1111/iej.13538. Epub 2021 May 28. PMID: 33908039.	Unrelated
7	Florou P, Anagnostis P, Theocharis P, Chourdakis M, Goulis DG. Does coenzyme Q10 supplementation improve fertility outcomes in women undergoing assisted reproductive technology procedures? A systematic review and meta-analysis of randomized-controlled trials. J Assist Reprod Genet. 2020 Oct;37(10):2377-2387. doi: 10.1007/s10815-020-01906-3. Epub 2020 Aug 7. PMID: 32767206; PMCID: PMC7550497.	No clinical measures
8	Tefiku U, Popovska M, Cana A, Zendeli-Bedxeti L, Recica B, Spasovska-Gjorgovska A, Spasovski S. Determination of the Role of Fusobacterium Nucleatum in the Pathogenesis in and Out the Mouth. Pril (Makedon Akad Nauk Umet Odd Med Nauki). 2020 Jun 1;41(1):87-99. doi: 10.2478/prilozi-2020-0026. PMID: 32573481.	Unrelated
9	Matei A, Saccone G, Vogel JP, Armson AB. Primary and secondary prevention of preterm birth: a review of systematic reviews and ongoing randomized controlled trials. Eur J Obstet Gynecol Reprod Biol. 2019 May;236:224-239. doi: 10.1016/j.ejogrb.2018.12.022. Epub 2019 Jan 25. PMID: 30772047.	Umbrella Review
10	Bett JVS, Batistella EÂ, Melo G, Munhoz EA, Silva CAB, Guerra ENDS, Porporatti AL, De Luca Canto G. Prevalence of oral mucosal disorders during pregnancy: A systematic review and meta-analysis. J Oral Pathol Med. 2019 Apr;48(4):270-277. doi: 10.1111/jop.12831. Epub 2019 Feb 12. PMID: 30673134.	No clinical measures
11	Abou El Fadl R, Blair M, Hassounah S. Integrating Maternal and Children's Oral Health Promotion into Nursing and Midwifery Practice- A Systematic Review. PLoS One. 2016 Nov 23;11(11):e0166760. doi: 10.1371/journal.pone.0166760. PMID: 27880790; PMCID: PMC5120808.	No clinical measures
12	Vanterpool SF, Tomsin K, Reyes L, Zimmermann LJ, Kramer BW, Been JV. Risk of adverse pregnancy outcomes in women with periodontal disease and the effectiveness of interventions in decreasing this risk: protocol for systematic overview of systematic reviews. Syst Rev. 2016 Feb 1;5:16. doi: 10.1186/s13643-016-0195-7. PMID: 26832150; PMCID: PMC4735974.	Systematic review protocol
13	Jiang H, Xiong X. Periodontitis may be Associated with Gestational Diabetes Mellitus but not Affirmatively. J Evid Based Dent Pract. 2016 Jun;16(2):121-3. doi: 10.1016/j.jebdp.2016.06.001. Epub 2016 Jun 4. PMID: 27449842.	Commentary
14	Vamos CA, Thompson EL, Avendano M, Daley EM, Quinonez RB, Boggess K. Oral health promotion interventions during pregnancy: a systematic review. Community Dent Oral Epidemiol. 2015 Oct;43(5):385-96. doi: 10.1111/cdoe.12167. Epub 2015 May 8. PMID: 25959402.	Unrelated
15	Gambhir RS, Nirola A, Gupta T, Sekhon TS, Anand S. Oral health knowledge and awareness among pregnant women in India: A systematic review. J Indian Soc	Unrelated

	Periodontol. 2015 Nov-Dec;19(6):612-7. doi: 10.4103/0972-124X.162196. PMID: 26941509; PMCID: PMC4753703.	
16	López NJ, Uribe S, Martinez B. Effect of periodontal treatment on preterm birth rate: a systematic review of meta-analyses. Periodontol 2000. 2015 Feb;67(1):87-130. doi: 10.1111/prd.12073. PMID: 25494599.	Umbrella review
17	Schwendicke F, Karimbux N, Allareddy V, Gluud C. Periodontal treatment for preventing adverse pregnancy outcomes: a meta- and trial sequential analysis. PLoS One. 2015 Jun 2;10(6):e0129060. doi: 10.1371/journal.pone.0129060. PMID: 26035835; PMCID: PMC4452791.	Data from a previous systematic review
18	Condylis B, Le Borgne H, Demoersman J, Campard G, Philippe HJ, Soueidan A. Intérêt du dépistage et du traitement des maladies parodontales chez la femme enceinte: revue de la littérature [Interest of periodontitis screening and treatment in pregnancy: systematic review]. J Gynecol Obstet Biol Reprod (Paris). 2013 Oct;42(6):511-7. French. doi: 10.1016/j.jgyn.2012.05.012. Epub 2012 Jun 27. PMID: 22743065.	Umbrella review
19	Niederman R. Pregnancy gingivitis and causal inference. Evid Based Dent. 2013 Dec;14(4):107-8. doi: 10.1038/sj.ebd.6400966. PMID: 24357820.	Commentary
20	Figuero E, Carrillo-de-Albornoz A, Martín C, Tobías A, Herrera D. Effect of pregnancy on gingival inflammation in systemically healthy women: a systematic review. J Clin Periodontol. 2013 May;40(5):457-73. doi: 10.1111/jcpe.12053. PMID: 23557432.	Unrelated
21	Michalowicz BS, Gustafsson A, Thumbigere-Math V, Buhlin K. The effects of periodontal treatment on pregnancy outcomes. J Clin Periodontol. 2013 Apr;40 Suppl 14:S195-208. doi: 10.1111/jcpe.12081. PMID: 23627329.	Review
22	Xiong X, Buekens P, Goldenberg RL, Offenbacher S, Qian X. Optimal timing of periodontal disease treatment for prevention of adverse pregnancy outcomes: before or during pregnancy? Am J Obstet Gynecol. 2011 Aug;205(2):111.e1-6. doi: 10.1016/j.ajog.2011.03.017. Epub 2011 Mar 16. PMID: 21620355.	Data from a previous systematic review
23	Ishaque S, Yakoob MY, Imdad A, Goldenberg RL, Eisele TP, Bhutta ZA. Effectiveness of interventions to screen and manage infections during pregnancy on reducing stillbirths: a review. BMC Public Health. 2011 Apr 13;11 Suppl 3(Suppl 3):S3. doi: 10.1186/1471-2458-11-S3-S3. PMID: 21501448; PMCID: PMC3231903.	Review
24	Polyzos NP, Polyzos IP, Zavos A, Valachis A, Mauri D, Papanikolaou EG, Tzioras S, Weber D, Messinis IE. Obstetric outcomes after treatment of periodontal disease during pregnancy: systematic review and meta-analysis. BMJ. 2010 Dec 29;341:c7017. doi: 10.1136/bmj.c7017. PMID: 21190966; PMCID: PMC3011371.	Commentary
25	George A, Johnson M, Blinkhorn A, Ellis S, Bhole S, Ajwani S. Promoting oral health during pregnancy: current evidence and implications for Australian midwives. J Clin Nurs. 2010 Dec;19(23-24):3324-33. doi: 10.1111/j.1365-2702.2010.03426.x. Epub 2010 Oct 19. PMID: 20955483.	Unrelated
26	Honest H, Forbes CA, Durée KH, Norman G, Duffy SB, Tsourapas A, Roberts TE, Barton PM, Jowett SM, Hyde CJ, Khan KS. Screening to prevent spontaneous preterm birth: systematic reviews of accuracy and effectiveness literature with economic modelling. Health Technol Assess. 2009 Sep;13(43):1-627. doi: 10.3310/hta13430. PMID: 19796569.	Unrelated
27	Menezes EV, Yakoob MY, Soomro T, Haws RA, Darmstadt GL, Bhutta ZA. Reducing stillbirths: prevention and management of medical disorders and infections during pregnancy. BMC Pregnancy Childbirth. 2009 May 7;9 Suppl 1(Suppl 1):S4. doi: 10.1186/1471-2393-9-S1-S4. PMID: 19426467; PMCID: PMC2679410.	Review
28	Manau C, Echeverria A, Agueda A, Guerrero A, Echeverria JJ. Periodontal disease definition may determine the association between periodontitis and pregnancy outcomes. J Clin Periodontol. 2008 May;35(5):385-97. doi: 10.1111/j.1600-051X.2008.01222.x. Epub 2008 Mar 12. PMID: 18341599.	Unrelated
29	Nugent JL, Baker PN. Periodontal disease and adverse pregnancy outcomes: a systematic review. BJOG. 2006 Jul;113(7):848; author reply 848-9. doi: 10.1111/j.1471-0528.2006.00969.x. Epub 2006 Jun 2. PMID: 16753047.	Commentary

Supplementary data 3. Characteristics of the included studies.

Author (Year), Country	Journal Type	Search Period	Guideline	ROB tool(s)	APOs	Periodontal outcome	N of included studies	ES	ES (95% CI)	ES type	MA Evidence	Random-Effects ($P< 10^{-6}$)	Random-Effects ($P< 10^{-3}$)	95% CI excluded the null value	I^2	FSN	AMSTAR Score
Merchant et al. (2022) [1], Columbia	Medical Journal | Reproduction	From October 2016 to March 2022	PRISMA	ROB-2	PTB (<37 weeks); LBW (<2,500 g)	Periodontal treatment	12; 8	RR	0.77 (0.58; 1.03); 0.66 (0.47; 0.93)	Binary	Suggestive; Weak	No; No	Yes; No	Yes; Yes	79,98; 56,51	3; 10	Critically Low
Zhang et al. (2022 [2], China	Medical Journal | General	Up to December 2020	PRISMA	NOS	PTB (<37 weeks); LBW (<2,500 g); SGA	Periodontal disease	15; 14; 4	OR	1.57 (1.39; 1.77); 2.39 (1.69; 3.38); 1.62 (0.8.6; 3.07)	Binary	Suggestive; Suggestive; Non-significant	No; No; No	Yes; Yes; No	Yes; Yes; No	7,9; 82,1; 57,5	278; 112	Critically Low
Le et al. (2022) [3], Australia / Canada / Vietnam	Dental Journal	Up to June 2020	PRISMA	Cochrane Collaboration's tool	PTB (<37 weeks); LBW (<2,500 g)	Periodontal treatment	11; 9; 3; 2	RR; OR; SMD	0.86 (0.66;1.13); 0.80 (0.58;1.10); 0.44 (0.20;0.98); 105.36 (36.72;174.01)	Binary; Binary; Binary; Continuous	Non-significant; Non-significant; Weak; Weak	No	No; No; No; Yes	No; No; Yes; Yes	49,94; 49,94; 36,1; 0	-	Critically Low
Porto et al. (2021) [4], Brazil	Medical Journal | General	Up to April 6, 2019	MOOSE	NOS	LBW <2,500 g	Periodontal disease	21; 16; 11	OR	2.13 (1.60;2.83)	Binary	Highly suggestive	Yes	Yes	Yes	80,8	188; 126; 211	Critically Low
Orlandi et al. (2021) [5], United Kingdom	Dental Journal	From 1946 to April 23, 2020	PRISMA	ROB-2	PTB (<37 weeks; <35 weeks; and <32 weeks); LBW (<2,500 g; and <1,500); PTB/LBW; Pre-eclampsia; SGA; Stillbirths; Perinatal mortality; Gestational age at delivery; Mean Birthweight	Periodontal treatment	14; 4; 2; 11; 3; 3; 4; 2; 6; 8; 3; 6	RR; SMD	0.77 (0.60;0.98); 0.89 (0.74;1.07); 0.83 (0.41;1.67); 0.77 (0.57;1.02); 1.02 (0.52;2.00); 0.39 (0.12;1.28); 1.00	Binary; Binary; Binary; Binary; Binary; Binary; Binary; Binary; Binary; Binary; Continuous; Continuous	Weak; Non-significant; Non-significant; Non-significant; Non-significant; Non-significant; Non-significant; Non-	No	No	Yes; No; No; No; No; No; No; No; No; No; No; No	67,1; 0; 55,3; 57,2; 47,6; 82,1; 6,4; 15,1; 9; 14,1; 15,1; 81,6	8	Critically Low

									(0.77;1.31); 0.90 (0.71;1.13); 0.64 (0.36;1.14); 0.85 (0.55;1.32); 0.35 (-0.23;0.93); 0.14 (-0.17;0.45)		significant; Non-significant; Non-significant; Non-significant; Non-significant						
Moliner-Sánchez et al. (2020) [6], Spain	Medical Journal | General	Up to May 6, 2020	PRISMA	NOS	PTB (<37 weeks); LBW (<2,500 g)	Periodontal disease	11; 6; 20	RR; OR	1.67 (1.17;2.38); 2.54 (1.61;3.98); 2.01 (1.71;2.36)	Binary	Highly suggestive; Highly suggestive; Strong	Yes	Yes	Yes; Yes; Yes	78,2; 59,72; 21,63	22; 30; 510	Critically Low
Konopka et al. (2020) [7], Poland	Medical Journal | Reproduction	Until the end of March 2019	PRISMA	NR	Pre-eclampsia	Periodontal disease	9	NA	NA	NA	NA	NA	NA	NA	NA	NA	Critically Low
Manrique-Corredor et al. (2019) [8], Colombia / Spain	Dental Journal	Up to November 2016	PRISMA	Critical Appraisal Skills Program CASPe and NOS	PTB (<37 weeks)	Periodontal disease	20	OR	2.01 (1.71;2.36)	Binary	Strong	Yes	Yes	Yes	21,63	510	Critically Low
Bi et al. (2019) [9], Canada	Medical Journal | Reproduction	Up to August 12, 2019	PRISMA	Cochrane Collaboration's tool	Perinatal mortality; PTB (<37 weeks); LBW (<2,500 g)	Periodontal treatment	8; 8; 8	OR; RR; Mean Difference	0.53 (0.30;0.93); 0.78 (0.62;0.98); 200.79 (64.34;337.24)	Binary; Binary; Continuous	Weak; Weak; Suggestive	No	No, No, Yes	Yes; Yes; Yes	0; 72; 93	7; 6; 17	Critically Low
Iheozor-Ejiofor et al. (2017) [10], United Kigdom	Medical Journal | General	Up to October 2016	Cochrane Handbook	Cochrane Collaboration's tool	PTB (<37 weeks); LBW (<2,500 g); Perinatal mortality; Pre-eclampsia	Periodontal treatment	11; 7; 7; 7	RR	0.87 (0.70;1.10); 0.67 (0.48;0.95); 0.85 (0.51;1.43); 1.10 (0.74;1.62)	Binary	Non-significant; Non-significant; Non-significant; Non-significant	No	No	No; No; No; No	66; 59; 21; 27	-	High

Silva et al.(2017) [11], Brazil / Canada	Medical Journal | General	Up to June 5, 2017	PRISMA	Cochrane Collaboration's tool	PTB (<37 weeks); LBW (<2.500g); Pre-eclampsia	Periodontal treatment	4; 2	RR	0.54 (0.38;0.77); 0.78 (0.5;1.21)	Binary	Weak; Non-significant	No	Yes, No	Yes; No	32; 41	14	Critically Low
Abariga et al. (2016) [12], USA	Medical Journal | Reproduction	April 2015 and updated on March 2016	PRISMA	NOS	GDM	Periodontal disease	10; 10	OR; OR adjusted	1.66 (1.17;2.36); 2.08 (1.21;3.58)	Binary	Weak; Suggestive	No	Yes	Yes; Yes	50,5; 36,9	20; 16	Critically Low
Corbella et al. (2016) [13], Germany	Medical Journal | General	From 1965 to January 30, 2015	NR	Cochrane Collaboration's tool	PTB (<37 weeks); LBW (<2,500 g); PTB/LBW	Periodontal disease	17; 10; 4	RR	1.61 (1.33;1.95); 1.65 (1.27;2.14); 3.44 (1.34;8.80)	Binary	Highly suggestive; Highly suggestive; Highly suggestive	Yes	Yes	Yes; Yes; Yes	79; 79; 90	133; 42; 6	Low
Esteves Lima et al. (2016) [14], Brazil	Dental Journal	July 2014 and updated on April 2015	PRISMA	NOS	GDM	Periodontal disease	4; 4	OR	1.67 (1.20;2.32); 1.69 (0.68; 4.21)	Binary	Strong; Non-significant	Yes; No	Yes; No	Yes; No	0; 81,37	10	Moderate
Teshome et al. (2016) [15], Ethiopia	Medical Journal | General	From January 2005 to December 2015	NR	NIH	PTB (<37 weeks); LBW (<2.500g)	Periodontal disease	10	NA	NA	NA	NA	NA	NA	NA	NA	NA	Critically Low
Huang et al. (2014) [16], China	Medical Journal | General	Up to March 2013	NR	MOOSE	Pre-eclampsia	Periodontal disease	11	NA	NA	NA	NA	NA	NA	NA	NA	NA	Low
Ide et al. (2013) [17], USA	Dental Journal	Up to May 2012	PRISMA	NOS	PTB (<37 weeks); BW; LBW (<2.500g); GA; SGA; Pre-eclampsia; PL/M	Periodontal disease	18	NA	NA	NA	NA	NA	NA	NA	NA	NA	Critically Low

Shah et al. (2013) [18], India	Medical Journal | Reproduction	From January 2000 to October 2012	NR	Cochrane Collaboration's tool	PTB (<37 weeks); LBW (<2.500g); PLBW	Periodontal treatment	13	NA	NA	NA	NA	NA	NA	NA	NA	NA	Critically Low
Stadelmann et al. (2013 [19], Switzerland	Dental Journal	Up to April 12, 2012	PRISMA	Developed by the authors	PTB; LBW; PLBW; Pre-eclampsia	Periodontal disease	8	NA	NA	NA	NA	NA	NA	NA	NA	NA	Critically Low
Boutin et al. (2013) [20], United Kingdom	Medical Journal | Reproduction	Up to February 2012	PRISMA	Cochrane Collaboration's tool	PTB (<37 weeks)	Periodontal treatment	12	RR	0.89 (0.73;1.08)	Binary	Non-significant	No	No	No	52	-	Critically Low
Sgolastra et al. (2013) [21], Italy	Medical Journal | General	Up to March 24, 2013	PRISMA	NOS	Pre-eclampsia	Periodontal disease	15	RR	2.17 (1.38;3.41)	Binary	Suggestive	No	Yes	Yes	78	48	High
Wei et al. (2013) [22], China	Medical Journal | General	Up to January 12, 2013	NR	NOS	Pre-eclampsia	Periodontal disease	15	RR	2.79 (2.01;3.01)	Binary	Highly suggestive	Yes	Yes	Yes	69,75	194	Critically Low
Rosa et al. (2012) [23], Brazil	Medical Journal | General	From 1980 to March 2012	PRISMA	Developed by the authors	PTB (<37 weeks); LBW (<2,500 g)	Periodontal disease	13; 9	RR	0.90 (0.68;1.19); 0.92 (0.71;1.20)	Binary	Non-significant; Non-significant	No	No	No; No	74; 56	-	Critically Low
Corbella et al. (2012) [24], Italy	Dental Journal	From 1965 to January 31, 2011	NR	NR	PTB (<37 weeks); LBW (<2,500 g); PTB/LBW	Periodontal disease	14; 7; 3	OR	1.78 (1.58;2.01); 1.82 (1.51;2.20); 3.00 (1.93;4.68)	Binary	Highly suggestive; Highly suggestive; Highly suggestive	Yes	Yes	Yes; Yes; Yes	82; 66; 89	452; 96; 24	Critically Low
Kim et al. (2012) [25], USA	Dental Journal	Up to September 19, 2011	PRISMA	Cochrane Collaboration's tool	PTB (<37 weeks); LBW (<2,500 g); Mean birthweight	Periodontal treatment	11; 8; 8	RR; Mean Difference	0.81 (0.64;1.02); 0.72 (0.48;1.07); 68.29 (-22.11;158.69)	Binary; Binary; Continuous	Non-significant; Non-significant; Non-significant	No	No	No; No; No	59; 75; 80	-	Critically Low

Konopka et al. (2012) [26], Poland	Medical Journal | Reproduction	From 1996 to 2010	NR	Margetts et al. 1995	PTB (<37 weeks); LBW (<2,500 g)	Periodontal disease	22	NA	NA	NA	NA	NA	NA	NA	NA	NA	Critically Low
Chambrone et al. (2011) I [27], Brazil	Dental Journal	Up to October 2010	Cochrane Handbook & PRISMA	NOS	PTB (<37 weeks); LBW (<2,500 g); PTB/LBW	Periodontal disease	7; 4; 5	RR	1.70 (1.03;2.81); 2.11 (1.05;4.23); 3.57 (1.87;6.84)	Binary	Weak; Weak; Highly suggestive	No; No; Yes	No; No; Yes	Yes; Yes; Yes	89; 86; 61	5; 3; 23	High
Chambrone et al. (2011) II [28], Brazil	Dental Journal	Up to October 2010	Cochrane Handbook, PRISMA & MOOSE	Cochrane Collaboration's tool	PTB (<37 weeks; <35 weeks; and <32 weeks); LBW (<2,500 g)	Periodontal treatment	11; 11; 5; 2; 3	RR	0.88 (0.72;1.09); 1.05 (0.84;1.30); 0.98 (0.73;1.31); 0.62 (0.36;1.08); 1.07 (0.86;1.33)	Binary	Non-significant; Non-significant; Non-significant; Non-significant; Non-significant	No	No	No; No; No; No; No	61; 46; 42; 0; 5	-	High
Fogacci et al. (2011) [29], Brazil	Medical Journal | Reproduction	Up to May 2010	Cochrane Handbook	Cochrane Collaboration's tool	PTB; LBW	Periodontal treatment	10	NA	NA	NA	NA	NA	NA	NA	NA	NA	High
George et al. (2011) [30], Australia	Medical Journal | General	Up to 2010	NR	JBI criteria	PTB (<37 weeks); LBW (<2,500 g); Spontaneous Abortion/Stillbirths	Periodontal treatment	10; 7; 8	OR	0.65 (0.45;0.93); 0.53 (0.31;0.92); 0.71 (0.43;1.16)	Binary	Highly suggestive; Suggestive; Non-significant	Yes; No; No	Yes; Yes; No	Yes; Yes; No	66; 69; 14	10; 7	Critically Low
Kunnen et al. (2010) [31], Netherlands	Dental Journal	Up to August 2010	NR	Cochrane Collaboration's tool	Pre-eclampsia	Periodontal disease	3	RR	1.00 (0.78;1.28)	Binary	Non-significant	No	No	No	51	-	Critically Low
Pimentel Lopes De Oliveira et al. (2010) [32], Brazil	Dental Journal	From November 1998 to October 2009	NR	NR	PTB (<37 weeks); LBW (<2.500g)	Periodontal treatment	7	NA	NA	NA	NA	NA	NA	NA	NA	NA	Critically Low

Uppal et al. (2010) [33], USA	Dental Journal	Up to March 2010	NR	Cochrane Collaboration's tool	PTB (<37 weeks)	Periodontal treatment	10	OR	0.59 (0.40;0.88)	Binary	Highly suggestive	Yes	Yes	Yes	75,8	17	Critically Low
Polyzos et al. (2009) [34], Greece	Medical Journal | Reproduction	Up to Januar y 2008	NR	NR	PTB (<37 weeks); LBW (<2,500 g); Spontaneous Abortion/Stillbirths	Periodontal treatment	7; 5; 6	OR	0.55 (0.35; 0.86); 0.48 (0.23;1.00); 0.73 (0.41;1.31)	Binary	Suggestive; Suggestive; Non-significant	No	Yes	Yes; Yes; No	50,7; 57,6; 22,2	11; 3	Critically Low
Conde-Agudelo et al. (2008) [35], USA	Medical Journal | Reproduction	Up to June 30, 2007	MOOSE	Previously reported classifications (Downs & Black, 1998; Levine, Walter, Lee, Haines, Holbrook & Moyer, 1994)	Pre-eclampsia	Periodontal disease	6	OR	2.37 (1.36;4.14)	Binary	Highly suggestive	Yes	Yes	Yes	80	15	Critically Low
Rustveld et al. (2008) [36], USA	Medical Journal | Reproduction	From 1964 to August 2006	MOOS E	Previously reported classifications	Pre-eclampsia	Periodontal disease	3	NA	NA	NA	NA	NA	NA	NA	NA	NA	Critically Low
Vergnes et al. (2007) [37], France	Medical Journal | Reproduction	Up to Septem ber 2005	MOOS E	Margetts et al. 1995	PTB; LBW	Periodontal disease	17	NA	NA	NA	NA	NA	NA	NA	NA	NA	Critically Low
Xiong et al. (2007) [38], USA	Medical Journal | Reproduction	From Januar y 1966 to Decem ber 2006	NR	NR	PTB (<37 weeks); BW; LBW (<2.500g); GA; SGA; GDM; Pre-eclampsia	Periodontal disease	44	NA	NA	NA	NA	NA	NA	NA	NA	NA	Critically Low
Vettore et al. (2006) [39], Brazil	Medical Journal | General	Up to Decem ber 21, 2005	NR	NR	PTB; LBW	Periodontal disease	36	NA	NA	NA	NA	NA	NA	NA	NA	NA	Critically Low
Xiong et al. (2006) [40], USA	Medical Journal | Reproduction	From Januar y 1966	NR	NR	PTB (<37 weeks); BW; LBW (<2.500g); GA;	Periodontal disease	25	NA	NA	NA	NA	NA	NA	NA	NA	NA	Critically Low

		to March 2005			SGA; GDM; Pre-eclampsia												
Khader et al. (2005) [41], Jordan	Dental Journal	From 1966 to August 2002	NR	Margetts et al. 1995	PTB; LBW	Periodontal disease	5	NA	NA	NA	NA	NA	NA	NA	NA	NA	Critically Low
Scannapieco et al. (2003) [42], USA	Dental Journal	Up to October 1, 2002	NR	Previously reported classifications	PTB; LBW	Periodontal disease	12	NA	NA	NA	NA	NA	NA	NA	NA	NA	Critically Low
Madianos et al. (2003) [43], USA	Dental Journal	Up to October 2001	Cochrane Handbook	NR	PTB; LBW	Periodontal disease	3	NA	NA	NA	NA	NA	NA	NA	NA	NA	Critically Low

Abbreviations: AMSTAR - A MeaSurement Tool to Assess Systematic Reviews; APOs – Adverse Pregnancy Outcomes; CI – confidence interval; ES – Effect Size; FSN – Fail-Safe Number; GA – Gestational age; GDM – Gestational Diabetes Mellitus; LBW – Low Birth Weight; MA – Meta-analysis; NA – Not Applicable; NOS – Newcastle-Ottawa Scale; NR – Not Reported; OR – Odds Ratio; PL/M - pregnancy loss or miscarriage; PLBW – Preterm Low Birth Weight; PTB – Preterm Birth; PTB/LBW - Preterm Birth/ Low Birth Weight; ROB – Risk of Bias; RR – Risk Ratio; SGA – Small for Gestational Age; SMD – Standardized Mean Difference

References

1. Merchant, A.T.; Gupta, R.D.; Akonde, M.; Reynolds, M.; Smith-Warner, S.; Liu, J.; Tarannum, F.; Beck, J.; Mattison, D. Association of Chlorhexidine Use and Scaling and Root Planing With Birth Outcomes in Pregnant Individuals With Periodontitis: A Systematic Review and Meta-Analysis. *JAMA Netw Open* **2022**, *5*, e2247632, doi:10.1001/jamanetworkopen.2022.47632.
2. Zhang, Y.; Feng, W.; Li, J.; Cui, L.; Chen, Z.-J. Periodontal Disease and Adverse Neonatal Outcomes: A Systematic Review and Meta-Analysis. *Front. Pediatr.* **2022**, *10*, 799740, doi:10.3389/fped.2022.799740.
3. Le, Q.-A.; Eslick, G.D.; Coulton, K.M.; Akhter, R.; Lain, S.; Nassar, N.; Yaacoub, A.; Condous, G.; Leonardi, M.; Eberhard, J.; et al. Differential Impact of Periodontal Treatment Strategies during Pregnancy on Perinatal Outcomes: A Systematic Review and Meta-Analysis. *Journal of Evidence-Based Dental Practice* **2022**, *22*, 101666, doi:10.1016/j.jebdp.2021.101666.
4. Porto, E.C.L.; Gomes Filho, I.S.; Batista, J.E.T.; Lyrio, A.O.; Souza, E.S.; Figueiredo, A.C.M.G.; Pereira, M.G.; Cruz, S.S. da Periodontite materna e baixo peso ao nascer: revisão sistemática e metanálise. *Ciênc. saúde coletiva* **2021**, *26*, 5383–5392, doi:10.1590/1413-812320212611.3.32362019.
5. Orlandi, M.; Muñoz Aguilera, E.; Marletta, D.; Petrie, A.; Suvan, J.; D'Aiuto, F. Impact of the Treatment of Periodontitis on Systemic Health and Quality of Life: A Systematic Review. *J Clinic Periodontology* **2022**, *49*, 314–327, doi:10.1111/jcpe.13554.
6. Moliner-Sánchez, C.A.; Iranzo-Cortés, J.E.; Almerich-Silla, J.M.; Bellot-Arcís, C.; Ortolá-Siscar, J.C.; Montiel-Company, J.M.; Almerich-Torres, T. Effect of per Capita Income on the Relationship between Periodontal Disease during Pregnancy and the Risk of Preterm Birth and Low Birth Weight Newborn. Systematic Review and Meta-Analysis. *IJERPH* **2020**, *17*, 8015, doi:10.3390/ijerph17218015.
7. Konopka, T.; Zakrzewska, A. Periodontitis and Risk for Preeclampsia — a Systematic Review. *Ginekol Pol* **2020**, *91*, 158–164, doi:10.5603/GP.2020.0024.
8. Manrique-Corredor, E.J.; Orozco-Beltran, D.; Lopez-Pineda, A.; Quesada, J.A.; Gil-Guillen, V.F.; Carratala-Munuera, C. Maternal Periodontitis and Preterm Birth: Systematic Review and Meta-analysis. *Community Dent Oral Epidemiol* **2019**, *47*, 243–251, doi:10.1111/cdoe.12450.
9. Bi, W.G.; Emami, E.; Luo, Z.-C.; Santamaria, C.; Wei, S.Q. Effect of Periodontal Treatment in Pregnancy on Perinatal Outcomes: A Systematic Review and Meta-Analysis. *The Journal of Maternal-Fetal & Neonatal Medicine* **2021**, *34*, 3259–3268, doi:10.1080/14767058.2019.1678142.
10. Iheozor-Ejiofor, Z.; Middleton, P.; Esposito, M.; Glenny, A.-M. Treating Periodontal Disease for Preventing Adverse Birth Outcomes in Pregnant Women. *Cochrane Database of Systematic Reviews* **2017**, *2017*, doi:10.1002/14651858.CD005297.pub3.
11. da Silva, H.E.C.; Stefani, C.M.; de Santos Melo, N.; de Almeida de Lima, A.; Rösing, C.K.; Porporatti, A.L.; Canto, G.D.L. Effect of Intra-Pregnancy Nonsurgical Periodontal Therapy on Inflammatory Biomarkers and Adverse Pregnancy Outcomes: A Systematic Review with Meta-Analysis. *Syst Rev* **2017**, *6*, 197, doi:10.1186/s13643-017-0587-3.
12. Abariga, S.A.; Whitcomb, B.W. Periodontitis and Gestational Diabetes Mellitus: A Systematic Review and Meta-Analysis of Observational Studies. *BMC Pregnancy Childbirth* **2016**, *16*, 344, doi:10.1186/s12884-016-1145-z.
13. Corbella, S.; Taschieri, S.; Del Fabbro, M.; Francetti, L.; Weinstein, R.; Ferrazzi, E. Adverse Pregnancy Outcomes and Periodontitis: A Systematic

Review and Meta-Analysis Exploring Potential Association. *Quintessence International* **2016**, *47*, 193–204, doi:10.3290/j.qi.a34980.
14. Lima, R.P.E.; Cyrino, R.M.; de Carvalho, B. Association Between Periodontitis and Gestational Diabetes Mellitus: Systematic Review and Meta-Analysis. *Journal of Periodontology* **2015**, 16.
15. Teshome, A.; Yitayeh, A. Relationship between Periodontal Disease and Preterm Low Birth Weight: Systematic Review. *Pan Afr Med J* **2016**, *24*, doi:10.11604/pamj.2016.24.215.8727.
16. Huang, X.; Wang, J.; Liu, J.; Hua, L.; Zhang, D.; Hu, T.; Ge, Z. Maternal Periodontal Disease and Risk of Preeclampsia: A Meta-Analysis. *J. Huazhong Univ. Sci. Technol. [Med. Sci.]* **2014**, *34*, 729–735, doi:10.1007/s11596-014-1343-8.
17. Ide, M.; Papapanou, P.N. Epidemiology of Association between Maternal Periodontal Disease and Adverse Pregnancy Outcomes – Systematic Review. *Journal of Periodontology* **2013**, *84*, S181–S194, doi:10.1902/jop.2013.134009.
18. Shah, M.; Muley, A.; Muley, P. Effect of Nonsurgical Periodontal Therapy during Gestation Period on Adverse Pregnancy Outcome: A Systematic Review. *The Journal of Maternal-Fetal & Neonatal Medicine* **2013**, *26*, 1691–1695, doi:10.3109/14767058.2013.799662.
19. Stadelmann, P.; Alessandri, R.; Eick, S.; Salvi, G.E.; Surbek, D.; Sculean, A. The Potential Association between Gingival Crevicular Fluid Inflammatory Mediators and Adverse Pregnancy Outcomes: A Systematic Review. *Clin Oral Invest* **2013**, *17*, 1453–1463, doi:10.1007/s00784-013-0952-0.
20. Boutin, A.; Demers, S.; Roberge, S.; Roy-Morency, A.; Chandad, F.; Bujold, E. Treatment of Periodontal Disease and Prevention of Preterm Birth: Systematic Review and Meta-Analysis. *Amer J Perinatol* **2012**, *30*, 537–544, doi:10.1055/s-0032-1329687.
21. Sgolastra, F.; Petrucci, A.; Severino, M.; Gatto, R.; Monaco, A. Relationship between Periodontitis and Pre-Eclampsia: A Meta-Analysis. *PLoS ONE* **2013**, *8*, e71387, doi:10.1371/journal.pone.0071387.
22. Wei, B.-J.; Chen, Y.-J.; Yu, L.; Wu, B. Periodontal Disease and Risk of Preeclampsia: A Meta-Analysis of Observational Studies. *PLoS ONE* **2013**, *8*, e70901, doi:10.1371/journal.pone.0070901.
23. Rosa, M.I. da; Pires, P.D.S.; Medeiros, L.R.; Edelweiss, M.I.; Martínez-Mesa, J. Periodontal Disease Treatment and Risk of Preterm Birth: A Systematic Review and Meta-Analysis. *Cad. Saúde Pública* **2012**, *28*, 1823–1833, doi:10.1590/S0102-311X2012001000002.
24. Corbella, S.; Taschieri, S.; Francetti, L.; De Siena, F.; Del Fabbro, M. Periodontal Disease as a Risk Factor for Adverse Pregnancy Outcomes: A Systematic Review and Meta-Analysis of Case–Control Studies. *Odontology* **2012**, *100*, 232–240, doi:10.1007/s10266-011-0036-z.
25. Kim, A.J.; Lo, A.J.; Pullin, D.A.; Thornton-Johnson, D.S.; Karimbux, N.Y. Scaling and Root Planing Treatment for Periodontitis to Reduce Preterm Birth and Low Birth Weight: A Systematic Review and Meta-Analysis of Randomized Controlled Trials. *J Periodontol* **2012**, *83*, 12.
26. Tomasz, K.; Anna, P.-S. Periodontitis and Risk of Preterm Birth and Low Birthweight – a Meta-Analysis. *Ginekol Pol.* **2012**, 8.
27. Chambrone, L.; Guglielmetti, M.R.; Pannuti, C.M.; Chambrone, L.A. Evidence Grade Associating Periodontitis to Preterm Birth and/or Low Birth Weight: I. A Systematic Review of Prospective Cohort Studies: Periodontitis and Adverse Pregnancy Outcomes. *Journal of Clinical Periodontology* **2011**, *38*, 795–808, doi:10.1111/j.1600-051X.2011.01755.x.

28. Chambrone, L.; Pannuti, C.M.; Guglielmetti, M.R.; Chambrone, L.A. Evidence Grade Associating Periodontitis with Preterm Birth and/or Low Birth Weight: II. A Systematic Review of Randomized Trials Evaluating the Effects of Periodontal Treatment: Periodontitis and Adverse Pregnancy Outcomes. *Journal of Clinical Periodontology* **2011**, *38*, 902–914, doi:10.1111/j.1600-051X.2011.01761.x.
29. Fogacci, M.F.; Vettore, M.V.; Thomé Leão, A.T. The Effect of Periodontal Therapy on Preterm Low Birth Weight: A Meta-Analysis. *Obstetrics & Gynecology* **2011**, *117*, 153–165, doi:10.1097/AOG.0b013e3181fdebc0.
30. George, A.; Shamim, S.; Johnson, M.; Ajwani, S.; Bhole, S.; Blinkhorn, A.; Ellis, S.; Andrews, K. Periodontal Treatment during Pregnancy and Birth Outcomes: A Meta-Analysis of Randomised Trials. *International Journal of Evidence-Based Healthcare* **2011**, *9*, 122–147, doi:10.1111/j.1744-1609.2011.00210.x.
31. Kunnen, A.; Van Doormaal, J.J.; Abbas, F.; Aarnoudse, J.G.; Van Pampus, M.G.; Faas, M.M. Periodontal Disease and Pre-Eclampsia: A Systematic Review: Periodontal Disease and Pre-Eclampsia. *Journal of Clinical Periodontology* **2010**, *37*, 1075–1087, doi:10.1111/j.1600-051X.2010.01636.x.
32. Pimentel Lopes De Oliveira, G.J.; Amaral Fontanari, L.; Chaves De Souza, J.A.; Ribeiro Costa, M.; Cirelli, J.A. Effect of Periodontal Treatment on the Incidence of Preterm Delivery: A Systematic Review. *Minerva Stomatol* **2010**, *59*, 543–550.
33. Uppal, A.; Uppal, S.; Pinto, A.; Dutta, M.; Shrivatsa, S.; Dandolu, V.; Mupparapu, M. The Effectiveness of Periodontal Disease Treatment During Pregnancy in Reducing the Risk of Experiencing Preterm Birth and Low Birth Weight. *The Journal of the American Dental Association* **2010**, *141*, 1423–1434, doi:10.14219/jada.archive.2010.0104.
34. Polyzos, N.P.; Polyzos, I.P.; Mauri, D.; Tzioras, S.; Tsappi, M.; Cortinovis, I.; Casazza, G. Effect of Periodontal Disease Treatment during Pregnancy on Preterm Birth Incidence: A Metaanalysis of Randomized Trials. *American Journal of Obstetrics and Gynecology* **2009**, *200*, 225–232, doi:10.1016/j.ajog.2008.09.020.
35. Conde-Agudelo, A.; Villar, J.; Lindheimer, M. Maternal Infection and Risk of Preeclampsia: Systematic Review and Metaanalysis. *American Journal of Obstetrics and Gynecology* **2008**, *198*, 7–22, doi:10.1016/j.ajog.2007.07.040.
36. Rustveld, L.O.; Kelsey, S.F.; Sharma, R. Association Between Maternal Infections and Preeclampsia: A Systematic Review of Epidemiologic Studies. *Matern Child Health J* **2008**, *12*, 223–242, doi:10.1007/s10995-007-0224-1.
37. Vergnes, J.-N.; Sixou, M. Preterm Low Birth Weight and Maternal Periodontal Status: A Meta-Analysis. *American Journal of Obstetrics and Gynecology* **2007**, *196*, 135.e1-135.e7, doi:10.1016/j.ajog.2006.09.028.
38. Xiong, X.; Buekens, P.; Vastardis, S.; Yu, S.M. Periodontal Disease and Pregnancy Outcomes: State-of-the-Science. *Obstetrical & Gynecological Survey* **2007**, *62*, 605–615, doi:10.1097/01.ogx.0000279292.63435.40.
39. Vettore, M.V.; Lamarca, G. de A.; Leão, A.T.T.; Thomaz, F.B.; Sheiham, A.; Leal, M. do C. Periodontal Infection and Adverse Pregnancy Outcomes: A Systematic Review of Epidemiological Studies. *Cad. Saúde Pública* **2006**, *22*, 2041–2053, doi:10.1590/S0102-311X2006001000010.
40. Xiong, X.; Buekens, P.; Fraser, W.; Beck, J.; Offenbacher, S. Periodontal Disease and Adverse Pregnancy Outcomes: A Systematic Review. *BJOG: Int J O&G* **2006**, *113*, 135–143, doi:10.1111/j.1471-0528.2005.00827.x.

41. Khader, Y.S.; Ta'ani, Q. Periodontal Diseases and the Risk of Preterm Birth and Low Birth Weight: A Meta-Analysis. *J Periodontol* **2005**, *76*, 5.
42. Scannapieco, F.A.; Bush, R.B.; Paju, S. Periodontal Disease as a Risk Factor for Adverse Pregnancy Outcomes. A Systematic Review. *Annals of Periodontology* **2003**, *8*, 70–78, doi:10.1902/annals.2003.8.1.70.
43. Madianos, P.N.; Bobetsis, G.A.; Kinane, D.F. Is Periodontitis Associated with an Increased Risk of Coronary Heart Disease and Preterm and/or Low Birth Weight Births?: Periodontitis and Systemic Disease. *Journal of Clinical Periodontology* **2002**, *29*, 22–36, doi:10.1034/j.1600-051X.29.s3.2.x.

Supplementary Data 4. AMSTAR 2 results.

N	#REF!	Review Quality	1	2	3	4	5	6	7	8	9	10	11	12	13	14	15	16
1	Merchant et al. (2022)	Critically Low	Y	PY	Y	N	Y	N	N	Y	Y/Y	Y	Y/Y	N	Y	Y	Y	Y
2	Zhang et al. (2022)	Critically Low	Y	Y	Y	N	Y	Y	N	Y	Y/Y	N	Y/Y	N	N	N	N	Y
3	Le et al. (2022)	Critically Low	Y	PY	Y	PY	Y	Y	N	Y	Y/Y	N	Y/Y	N	N	N	N	Y
4	Porto et al. (2021)	Critically Low	Y	Y	Y	N	Y	Y	N	N	Y/Y	N	Y/Y	Y	Y	Y	Y	N
5	Orlandi et al. (2021)	Critically Low	N	PY	Y	N	Y	Y	Y	Y	Y/Y	Y	Y/Y	N	Y	Y	Y	Y
6	Moliner-Sánchez et al. (2020)	Critically Low	Y	PY	Y	PY	Y	Y	N	Y	Y/Y	N	Y/Y	N	Y	Y	Y	Y
7	Konopka et al. (2020)	Critically Low	Y	N	Y	N	Y	Y	Y	Y	N/N	N	NA	NA	N	NA	NA	Y
8	Manrique-Corredor et al. (2019)	Critically Low	Y	PY	Y	N	Y	Y	N	Y	Y/Y	N	Y/Y	N	Y	Y	Y	Y
9	Bi et al. (2019)	Critically Low	Y	PY	Y	PY	Y	Y	N	Y	Y/Y	N	Y/Y	Y	Y	Y	Y	Y
10	Iheozor-Ejiofor et al. (2017)	High	Y	Y	Y	PY	Y	Y	Y	Y	Y/Y	Y	Y/Y	Y	Y	Y	Y	Y
11	Silva et al. (2017)	Critically Low	Y	PY	Y	PY	Y	Y	Y	Y	Y/Y	N	Y/Y	Y	Y	Y	Y	Y
12	Abariga et al. (2016)	Critically Low	Y	PY	Y	N	N	Y	N	Y	Y/Y	N	Y/Y	Y	Y	Y	Y	Y
13	Corbella et al. (2016)	Low	Y	PY	Y	PY	Y	Y	Y	Y	Y/Y	N	Y/Y	Y	Y	Y	Y	N
14	Esteves Lima et al. (2016)	Moderate	Y	Y	Y	PY	Y	Y	Y	Y	Y/Y	N	Y/Y	N	Y	Y	Y	Y
15	Teshome et al. (2016)	Critically Low	Y	PY	Y	N	Y	Y	N	Y	Y/Y	N	NA	NA	N	NA	NA	Y
16	Huang et al. (2014)	Low	Y	Y	Y	N	N	Y	Y	Y	Y/Y	N	Y/Y	Y	Y	Y	Y	Y
17	Ide et al. (2013)	Critically Low	Y	N	Y	PY	Y	Y	N	Y	N/N	N	NA	NA	N	NA	NA	Y
18	Shah et al. (2013)	Critically Low	Y	PY	Y	N	Y	Y	Y	Y	Y/Y	N	NA	NA	N	NA	NA	Y
19	Stadelmann et al. (2013)	Critically Low	Y	N	Y	N	Y	Y	Y	Y	N/N	N	NA	NA	N	NA	NA	Y
20	Boutin et al. (2013)	Critically Low	Y	Y	Y	PY	Y	Y	N	Y	Y/Y	N	Y/Y	Y	Y	Y	Y	Y
21	Sgolastra et al. (2013)	High	Y	PY	Y	PY	Y	Y	Y	Y	Y/Y	N	Y/Y	Y	Y	Y	Y	Y
22	Wei et al. (2013)	Critically Low	Y	N	Y	PY	Y	Y	Y	Y	N/N	N	Y/Y	Y	Y	Y	Y	Y
23	Rosa et al. (2012)	Critically Low	Y	PY	Y	PY	Y	Y	N	Y	Y/Y	N	Y/Y	N	Y	Y	Y	N
24	Corbella et al. (2012)	Critically Low	Y	N	Y	N	Y	N	N	Y	N/N	N	Y/Y	N	N	N	N	N
25	Kim et al. (2012)	Critically Low	Y	Y	Y	PY	Y	Y	N	Y	Y/Y	N	Y/Y	N	Y	Y	Y	Y
26	Konopka et al. (2012)	Critically Low	Y	N	Y	N	N	N	N	Y	N/N	N	Y/Y	Y	Y	Y	Y	N
27	Chambrone et al. (2011) I	High	Y	PY	Y	Y	Y	Y	Y	Y	Y/Y	Y	Y/Y	N	Y	Y	Y	Y
28	Chambrone et al. (2011) II	High	Y	Y	Y	Y	Y	Y	Y	Y	Y/Y	Y	Y/Y	N	Y	Y	Y	Y
29	Fogacci et al. (2011)	High	Y	Y	Y	PY	Y	Y	Y	Y	Y/Y	N	Y/Y	Y	Y	Y	Y	Y
30	George et al. (2011)	Critically Low	Y	PY	Y	N	Y	Y	Y	Y	Y/Y	N	Y/Y	Y	Y	Y	Y	N
31	Kunnen et al. (2010)	Critically Low	Y	PY	Y	N	Y	N	Y	Y	Y/Y	N	Y/Y	Y	Y	Y	Y	Y
32	Pimentel Lopes De Oliveira et al. (2010)	Critically Low	Y	N	Y	N	N	N	Y	Y	N/N	N	NA	NA	N	NA	NA	N
33	Uppal et al. (2010)	Critically Low	Y	Y	Y	N	Y	N	N	Y	Y/Y	N	Y/Y	Y	Y	Y	Y	N

34	Polyzos et al. (2009)	Critically Low	Y	PY	Y	PY	Y	Y	N	Y	N/N	N	Y/Y	Y	Y	Y	Y	N
35	Conde-Agudelo et al. (2008)	Critically Low	Y	PY	Y	PY	N	N	N	Y	N/N	N	Y/Y	Y	Y	Y	Y	Y
36	Rustveld et al. (2008)	Critically Low	Y	PY	Y	N	N	N	Y	Y	N/N	N	Y/Y	N	Y	Y	Y	N
37	Vergnes et al. (2007)	Critically Low	Y	PY	Y	PY	Y	Y	N	Y	N/N	N	Y/Y	Y	Y	Y	Y	N
38	Xiong et al. (2007)	Critically Low	Y	N	Y	N	N	N	N	Y	N/N	N	Y/Y	N	N	N	N	Y
39	Vettore et al. (2006)	Critically Low	Y	N	Y	N	N	N	N	Y	N/N	N	NA	NA	N	NA	NA	N
40	Xiong et al. (2006)	Critically Low	Y	N	Y	N	N	N	N	Y	N/N	N	NA	NA	N	NA	NA	N
41	Khader et al. (2005)	Critically Low	Y	PY	Y	N	Y	N	Y	Y	N/N	N	Y/Y	Y	Y	Y	Y	N
42	Scannapieco et al. (2003)	Critically Low	Y	N	Y	N	Y	N	N	Y	N/N	N	NA	NA	N	NA	NA	N
43	Madianos et al. (2003)	Critically Low	Y	N	Y	N	Y	N	Y	Y	N/N	N	NA	NA	N	NA	NA	N

N—No, Y—Yes, PY—Partial Yes. 1. Research questions and inclusion criteria? 2. Review methods established a priori? 3. Explanation of their selection literature search strategy? 4. Did the review authors use a comprehensive literature search strategy? 5. Study selection performed in duplicate? 6. Data selection performed in duplicate? 7. List of excluded studies and exclusions justified? 8. Description of the included studies in adequate detail? 9. Satisfactory technique for assessing the risk of bias (RoB)? 10. Report on the sources of funding for the studies included in the review? 11. If meta-analysis was performed, did the review authors use appropriate methods for statistical combination of results? 12. If meta-analysis was performed, did the review authors assess the potential impact of RoB? 13. RoB accounted when interpreting/discussing the results of the review? 14. Did the review authors provide a satisfactory explanation for, and discussion of, any heterogeneity observed in the results of the review? 15. If they performed quantitative synthesis, was publication bias performed? 16. Did the review authors report any potential sources of conflict of interest, including funding sources?.

Supplementary Data 5. Overlap of study results across systematic reviews on the association of periodontal disease with APOs.

	Study Type	Scannapieco et al. (2003)	Madianos et al. (2003)	Khader et al. (2005)	Vettore et al. (2006)	Xiong et al. (2006)	Vergnes et al. (2007)	Xiong et al. (2007)	Conde-Agudelo et al. (2008)	Rustveld et al. (2008)	Kunnen et al. (2010)	Chambrone et al. (2011) I	Rosa et al. (2012)	Corbella et al. (2012)	Konopka et al. (2012)	Ide et al. (2013)	Stadelmann et al. (2013)	Sgolastra et al. (2013)	Wei et al. (2013)	Huang et al. (2014)	Abariga et al. (2016)	Corbella et al. (2016)	Esteves Lima et al. (2016)	Teshome et al. (2016)	Manrique-Corredor et al. (2019)	Moliner-Sánchez et al. (2020)	Konopka et al. (2020)	Porto et al. (2021)	Zhang et al. (2022)	**Overlap %**
Offenbacher et al. (1996)	C/C	X	X	X	X	X	X								X										X					28,57%
Offenbacher et al. (1998)	C/C	X	X		X												X													14,29%
Dasanayake et al. (1998)	C/C	X	X			X																								10,71%
Dasanayake et al. (2001)	C/C	X		X																										7,14%
Romero et al. (2002)	C/C	X				X																								7,14%
Dasanayake (2002)	C/C	X																												3,57%
Davenport et al. (2002)	C/C	X			X	X																								10,71%
Jeffcoat et al. (2001)	C		X	X	X	X						X			X															21,43%
Mitchell-Lewis et al. (2001)	C/C		X	X		X		X																						14,29%
Lopez et al. (2002)	CT			X	X	X			X				X													X				21,43%
Jeffcoat et al. (2003)	CT				X	X	X	X	X				X																	21,43%
Sembene et al. (2000)	C/C				X	X																								7,14%
Louro et al. (2001)	C/C				X	X	X																					X		14,29%
Cruz et al. (2005)	C/C				X																							X	X	10,71%
Madianos et al. (2001)	C/C				X																									3,57%
Hasegawa et al. (2003)	C/C				X																									3,57%
Moore et al. (2004) BDJ	C/C				X	X	X					X			X														X	21,43%
Moore et al. (2005)	C/C				X	X										X														10,71%
Goepfert et al. (2004)	C/C				X	X	X							X	X							X			X					25,00%
Jarjoura et al. (2005)	C/C				X	X	X							X	X	X						X			X					28,57%
Moore et al. (2004)	P/C				X																				X					7,14%
Moreu et al. (2005)	C/C				X																									3,57%
Lunardelli & Peres (2005)	C/C				X		X								X	X														14,29%
Cardoso (1999)	C/C				X																									3,57%
Mookem et al. (2004)	C/C				X	X	X																							10,71%
Radnai et al. (2004)	C/C				X	X	X							X								X								17,86%
Dörtbudak et al. (2005)	C/C				X	X	X								X															14,29%
Moliterno et al. (2005)	C/C				X		X								X									X						14,29%
Konopka et al. (2003)	C/C				X		X								X		X													14,29%
Carta et al. (2004)	C/C				X												X													7,14%
Buduneli et al. (2005)	C/C				X	X																								7,14%
Noack et al. (2005)	C/C				X		X								X		X													14,29%
Holbrook et al. (2004)	C				X	X																								7,14%
Marin et al. (2005)	C				X		X							X								X								14,29%

Offenbacher et al. (2001)	C				X	X						X																		10,71%
Rajapakse et al. (2005)	C				X	X	X					X			X															17,86%
Lopez et al. (2002)	CT				X	X		X						X	X							X								21,43%
Fraser et al. (unpublished data)	C/C					X																								3,57%
Canakci et al. (2004)	C/C					X			X	X	X						X	X	X	X										28,57%
Boggess et al. (2003)	C					X			X	X	X							X	X	X							X			28,57%
Glesse et al. (2004)	C						X																							3,57%
Robles et al. (2004)	C/C						X																							3,57%
Sadatmansouri et al. (2006)	CT							X					X																	7,14%
Michalowicz et al. (2006)	CT							X			X		X													X	X			17,86%
Oettinger et al. (2005)	C/C								X	X							X													10,71%
Contreras et al. (2006)	C/C								X	X	X								X	X										17,86%
Castaldi et al. (2006)	C								X		X								X											10,71%
Khader et al. (2006)	C/C								X		X																			7,14%
Cota et al. (2006)	C/C								X										X											7,14%
Kunnen et al. (2007)	C/C								X		X							X	X											14,29%
Barak et al. (2007)	C/C								X																					3,57%
Blaauw et al. (2005)	C									X																				3,57%
López et al. (2005)	CT												X																	3,57%
Offenbacher et al. (2006)	CT											X	X	X			X					X				X			X	25,00%
Tarannum and Faizuddin (2007)	CT												X																	3,57%
Canakci et al. (2007)	C/C										X							X	X											10,71%
Siqueira et al. (2008)	C/C										X				X	X		X	X	X		X			X			X		32,14%
Lohsoonthorn et al. (2009)	C/C										X							X	X	X										14,29%
Srinivas et al. (2009)	C										X	X						X								X	X			17,86%
Nabet et al. (2010)	C/C										X			X		X						X			X				X	21,43%
Shetty et al. (2010)	C/C										X							X	X	X							X			17,86%
Offenbacher et al. (2009)	CT										X		X														X			10,71%
Newnham et al. (2009)	CT										X		X														X			10,71%
Macones et al. (2010)	CT												X													X				7,14%
Oliveira et al. (2011)	CT												X																	3,57%
Agueda et al. (2008)	P/C											X		X	X							X			X				X	21,43%
Pitiphat et al. (2008)	P/C											X													X				X	10,71%
Rakoto-Alson et al. (2010)	C											X		X								X				X				14,29%
Saddki et al. (2008)	P/C											X														X		X	X	14,29%
Sharma et al. (2007)	C											X																		3,57%
Weidlich et al. (2012)	CT												X																	3,57%
Sant'Ana et al. (2011)	CT												X																	3,57%
Ryu et al. (2010)	C/C													X								X			X				X	14,29%
Guimarães et al. (2010)	C/C													X		X						X								10,71%
Vogt et al. (2010)	P/C													X								X				X		X	X	17,86%
Cruz et al. (2009)	C/C													X								X						X		10,71%
Radnai et al. (2006)	C/C													X	X	X						X			X					17,86%
Mafra-Siqueira et al. (2007)	C/C													X																3,57%

Wood et al. (2006)	C/C													X		X						X								10,71%
Mumghamba et al. (2007)	C/C													X								X		X	X					14,29%
Arteaga-Guerra et al. (2010)	C/C														X															3,57%
Betleja et al. (2004)	C/C														X															3,57%
Bosnjak et al. (2006)	C/C														X	X														7,14%
Bassani et al. (2007)	C/C														X													X		7,14%
Gomes-Filho et al. (2007)	C/C														X										X			X		10,71%
Marakoglu et al. (2008)	C/C														X															3,57%
Khader et al. (2009)	C/C														X									X						7,14%
Le et al. (2007)	C/C															X														3,57%
Santos-Pereira et al. (2007)	C															X														3,57%
Toygar et al. (2007)	C															X														3,57%
Heimonen et al. (2008)	C/C															X														3,57%
Vettore et al. (2008)	C/C															X														3,57%
Giannella et al. (2011)	C/C															X														3,57%
Iwanaga et al. (2011)	C															X														3,57%
Piscoya et al. (2012)	C/C															X														3,57%
Tarannum et al. (2011)	C-S																X													3,57%
Chaparro et al. (2012)	C/C																	X												3,57%
Ha et al. (2011)	C/C																	X	X	X										10,71%
Kumar et al. (2013)	P/C																	X		X					X		X	x	X	21,43%
Politano et al. (2011)	C/C																	X	X	X										10,71%
Moura da Silva et al. (2012)	C/C																	X	X	X										10,71%
Taghzouti et al (2012)	C/C																	X	X	X										10,71%
Wang et al. (2012)	C/C																	X												3,57%
Sayar et al. (2011)	C/C																		X											3,57%
Novak et al. (2006)	C/S																				X		X							7,14%
Chokwiriyachit et al (2013)	C/C																				X		X							7,14%
Xiong et al (2009)	C/C																				X		X							7,14%
Esteves−Lima et al (2013)	C/C																				X		X							7,14%
Bulllon P et al (2014)	C/S																				X		X							7,14%
Bagis N et al (2013)	C/C																				X									3,57%
Habib FA et al (2009)	C/C																				X		X							7,14%
Dasanayake et al (2008)	C/C																				X		X							7,14%
Ruiz et al (2011)	C/C																				X		X							7,14%
Mishra P et al (2014)	C/C																				X									3,57%
Guimarães et al (2012)	C/C																					X								3,57%
Santa Cruz et al (2012)	C/C																					X						X		7,14%
Ali et al (2012)	P/C																					X								3,57%
Abati et al (2013)	C																					X						X		7,14%
Ahmed et al. (2013)	C/C																							X						3,57%
Cisse et al. (2015)	C/C																							X				X		7,14%
Mannem et al. (2011)	C/C																							X						3,57%
Gandi et al. (2010)	C/C																							X						3,57%

Kukkamal et al. (2014)	C/C																						X						3,57%
Smitha et al. (2013)	C/C																						X						3,57%
Baskaradoss et al (2008)	C/C																											X	3,57%
Bassini et al (2007)	C/C																											X	3,57%
Bogges et al (2006)	P/C																											X	3,57%
Erchick et al. (2020)	P/C																											X	3,57%
Filho et al. (2016)	C/C																											X	3,57%
Jacob et al (2014)	C/C																										X	X	7,14%
Khan et al (2016)	C/C																										X	X	7,14%
Macedo et al (2014)	C/C																					X			X				X	10,71%
Mathew et al (2014)	C/C																										X	X	7,14%
Micu et al (2020)	P/C																											X	3,57%
Novak et al (2020)	C/C																											X	3,57%
Sousa et al (2016)	C/C																											X	3,57%
Tejada et al (2012)	C/C																								X				X	7,14%
Turton et al (2017)	P/C																											X	3,57%
Lafaurie et al. (2020)	C/C																										X		3,57%
Trindade et al (2016)	C																										X		3,57%
Pozo et al (2016)	C/C																										X		3,57%
Souza et al (2016)	C/C																										X		3,57%
Kumar et al (2018)	P/C																										X		3,57%
Komara et al (2016)	C/C																										X		3,57%
Ha et al (2014)	P/C																									X			3,57%
Soucy-Giguère et al (2016)	P/C																								X	X			7,14%
Pare et al (2009)	P/C																								X				3,57%
Tellapragada et al (2016)	P/C																								X				3,57%
Mokeem et al (2004)	C/C																							X					3,57%
Grandi et al (2009)	C/C																							X					3,57%
Baskaradoss et al (2011)	C/C																							X					3,57%
Bulut et al (2014)	C/C																							X					3,57%
Andonova et al (2015)	C/C																							X					3,57%

C – Cohort study; C/C – Case-control study; CT – Randomized Clinical Trials; P/C – Prospective/Cohort study.

Supplementary Data 6. Overlap of study results across systematic reviews on the periodontal treatment effect on APOs.

	Study Type	Polyzos et al. (2009)	PL De Oliveira et al. (2010)	Uppal et al. (2010)	Chambrone et al. (2011) II	Fogacci et al. (2011)	George et al. (2011)	Kim et al. (2012)	Shah et al. (2013)	Boutin et al. (2013)	Iheozor-Ejiofor et al. (2017)	Silva et al.(2017)	Bi et al. (2019)	Orlandi et al. (2021)	Le et al. (2022)	Merchant et al. (2022)	**Overlap %**
Lopez et al. (2002)	CT				X	X	X	X	X	X	X		X	X	X	X	73,33%
Jeffcoat et al. (2003)	CT			X	X	X	X	X	X	X	X		X				60,00%
Lopez et al. (2002)	CT			X											X		13,33%
Sadatmansouri et al. (2006)	CT	X	X		X	X	X	X	X	X	X		X	X	X	X	86,67%
Michalowicz et al. (2006)	CT	X	X	X	X	X	X	X	X	X	X		X		X	X	86,67%
López et al. (2005)	CT	X	X	X					X	X	X		X			X	53,33%
Offenbacher et al. (2006)	CT	X	X	X	X	X	X	X		X	X	X	X	X	X	X	93,33%
Tarannum and Faizuddin (2007)	CT	X	X	X	X	X	X	X	X	X	X		X	X	X	X	93,33%
Offenbacher et al. (2009)	CT			X	X	X	X	X	X	X	X		X	X	X		73,33%
Newnham et al. (2009)	CT			X	X	X	X	X	X	X	X		X	X	X	X	80,00%
Gazolla et al. (2007)	CT		X						X						X		20,00%
Radnai et al. (2009)	CT		X	X	X	X	X	X	X		X		X	X	X	X	80,00%
Novak et al. (2009)	CT												X		X		13,33%
Macones et al. (2010)	CT			X	X	X		X	X	X	X		X	X	X		66,67%
Deppe et al. (2010)	CT														X		6,67%
Jeffcoat et al. (2011)	CT				X			X					X	X	X		33,33%
Oliveira et al. (2011)	CT				X			X	X	X	X		X	X	X	X	60,00%
Pirie et al. (2013)	CT										X	X	X		X		26,67%
Reddy et al. (2014)	CT													X	X		13,33%
Khainar et al. (2015)	CT											X			X		13,33%
Jannaina et al. (2015)	CT														X		6,67%
Novak et al. (2018)	CT														X		6,67%
Leticia et al. (2019)	CT														X		6,67%
Weidlich et al. (2012)	CT								X	X			X				20,00%
Sant'Ana et al. (2011)	CT												X				6,67%
Farrell et al (2003)	CT										X		X			X	20,00%
Herera et al (2009)	CT										X		X			X	20,00%
Caneiro-Queija et al (2019)	CT													X		X	13,33%
Penova-Veselinovic et al. (2015)	P/C											X					6,67%
Khairnar et al (2015)	CT												X	X			13,33%
Michaelowicz et al (2006)	CT													X			6,67%

CT – Randomized Clinical Trials; P/C – Prospective/Cohort study
